# Design, measurement and processing of region-specific DNA methylation assays: the mass spectrometry-based method EpiTYPER

**DOI:** 10.3389/fgene.2015.00287

**Published:** 2015-09-17

**Authors:** H. Eka D. Suchiman, Roderick C. Slieker, Dennis Kremer, P. Eline Slagboom, Bastiaan T. Heijmans, Elmar W. Tobi

**Affiliations:** Molecular Epidemiology, Department of Medical Statistics and Bioinformatics, Leiden University Medical CenterLeiden, Netherlands

**Keywords:** bisulfite, EpiTYPER®, region-specific, mass spectrometry, quantitative, DNA methylation

## Abstract

EpiTYPER® is a mass spectrometry-based bisulfite sequencing method that enables region-specific DNA methylation analysis in a quantitative and high-throughput fashion. The technology targets genomic regions of 100–600 base pairs and results in the quantitative measurement of DNA methylation levels largely at single-nucleotide resolution. It is particularly suitable for larger scale efforts to study candidate regions or to validate regions from genome-wide DNA methylation studies. Here, we describe in detail how to design and perform EpiTYPER measurements and preprocess the data, providing details for high quality measurements not provided in the standard EpiTYPER protocol.

## Introduction

EpiTYPER® (Ehrich et al., 2005) is a technology to quantitatively assess DNA methylation of multiple CpGs in genomic regions of 100–600 base-pairs. EpiTYPER® uses the Agena Bioscience (previously Sequenom Inc.) MassArray® set-up which has a high degree of automation and sufficient throughput for the larger scale evaluation of candidate regions. EpiTYPER has been successfully used for candidate gene studies in many fields, including in studies on cancer (Radpour et al., 2009), development (Loke et al., 2013), and nutrition (do Amaral et al., 2014). Another major application is in the validation of regions identified in various genome-wide DNA methylation assays (Christensen et al., 2010; Figueroa et al., 2010; Popp et al., 2010; Zeilinger et al., 2013; Tobi et al., 2014) or follow-up of findings from such studies in thousands of additional samples (Zhang et al., 2014).

The good quantitative accuracy of the technology has been used to detect DNA methylation differences between conditions down to a few percent points depending on the sample size (Tobi et al., 2012). EpiTYPER is particularly useful for projects that require the measurement of larger numbers of samples or regions since a single EpiTYPER run yields 126 triplicate measurements. Including required controls, it entails an experiment on a 384-well PCR plate. The set-up is flexible in the number of samples and regions that are combined in a run. The method is, however, not cost-effective for projects entailing less than 126 triplicate measurements.

**Figure 1:**
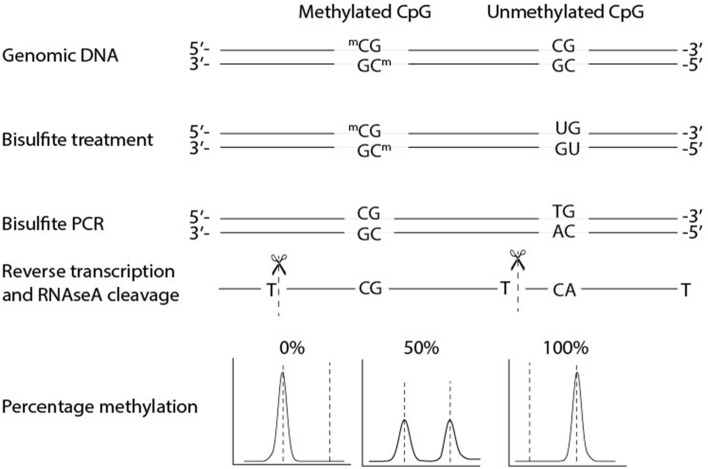
**Biochemical steps and methylation quantification with EpiTYPER®**. EpiTYPER® resolves on re-sequencing DNA on mass. After bisulfite conversion DNA is amplified by PCR and the resulting PCR product is transcribed from the reverse strand of the PCR product. This single stranded product is cleaved after each T resulting in fragments of known mass. Fragments with a methylated cytosine are 16 Dalton heavier than fragments with a non-methylated cytosine, the size of the two peaks can be used to calculate the methylation ratio of the CpG dinucleotide. It is easy to envision that fragments can arise with the same mass or with a mass extremely close to that of another fragment. Such circumstances limit the detection of certain CpG dinucleotides.

The principle of EpiTYPER is mass based re-sequencing of PCR amplified bisulfite converted DNA with a mass spectrometer and involves several biochemical steps (Figure 1) (Ehrich et al., 2005, 2007). In short, genomic DNA (gDNA) is treated with bisulfite which leads to a conversion of all unmethylated cytosines, while methylated cytosines remain unaffected. A PCR is performed on the bisulfite converted DNA with primers tagged with a T7 promoter. After shrimp alkaline phosphatase treatment, to discard unincorporated DNA nucleotides, the T7 promoter added during PCR is used to transcribe the PCR product from the reverse strand, yielding a single stranded RNA product. This RNA product is cleaved with RNase A resulting in a specific fragmentation of the RNA product. After a final cleaning step with a resin the samples are loaded on a SpectroCHIP® II Array, which prepares the fragments for separation on mass with the mass spectrometer. The mass spectrometer is a Matrix Assisted Laser Desorption-Ionization Time of Flight (MALDI-TOF) device. The matrix on the SpectroCHIP II absorbs the energy of the laser and transfers it to the RNA fragments which subsequently become ionized. The ionized fragments are separated by the time it takes to arrive at the detector in at the end of the mass spectrometer's flight tube under the influence of an electric field. The time of flight increases with higher mass. A fragment containing one or more CpG dinucleotides is called a CpG unit. If a CpG dinucleotide was methylated and protected from bisulfite conversion, the corresponding RNA fragment, the CpG unit, will be 16 Da heavier in mass when the CpG dinucleotide was methylated, resulting in a 16 Da shift in the mass spectrum. The signal detected by the mass spectrometer for either fragments is proportional to the number of fragments. The number of fragments is quantified by the surface area of the corresponding peaks in the mass spectrum. The DNA methylation percentage of a given CpG is calculated by dividing the surface area of the peak representing the methylated fragment by the total surface area of the peaks of both the methylated and unmethylated fragment.

For an effective application of EpiTYPER, a streamlined process of assay design, assay validation, bisulfite conversion, data generation, and data processing is necessary. These steps involve switching between lab and computer work. We will provide a step-wise description of all these steps and also include various tips. These steps are an addition to the protocol provided for EpiTYPER by Agena Biosciences.

## Materials and equipment

### Prerequisites

Agena Bioscience Inc. provides several automated pipelines for the measurement of nucleotides (SNPs, gene-expression etc.) on the MassARRAY® system. If a MassARRAY system is already available EpiTYPER only requires an additional proprietary software suite, and some additional fine-tuning of the MALDI-TOF mass spectrometer by a specialized service engineer to function. If a MassARRAY system is not yet available EpiTYPER will require a significant investment in hardware, including a compatible provided MALDI-TOF mass spectrometer, an automated liquid handling system to transfer samples from 384 plates to the SpectroCHIP II and a dedicated server to process and store the data generated.

### Consumables

#### Bisulfite conversion

1. EZ-96 DNA methylation kit, Shallow-Well Format (ZYMO Research) or for less samples the EZ DNA methylation kit (ZYMO Research). For a large number of samples we advise to use the 96-well format to reduce the number of batches in the experiment.
2. Tris base (ROCHE).
3. Ethanol absolute (molecular biology grade).

#### EpiTYPER

4. Ethylenediaminetetraacetic acid disodium salt dihydrate (Sigma-Aldrich).
5. Thermo-Fast 384 PCR Plates (Westburg), or plastics appropriate for your PCR set-up.
6. SpectroCHIP® II Array and Clean Resin Kit (Agena Bioscience Inc).
7. MassCLEAVE™, T Cleavage Kit (Agena Bioscience Inc).
8. Hotstar taq DNA polymerase (Qiagen, including buffer).
9. dNTP mix, PCR grade (Qiagen; 10 mM of each nucleotide).

#### Solutions to prepare

1. 1M Tris-HCl pH7.5 (1L): add 141.1 g Tris base to 800 ml ddH20, adjust pH by adding approximately 60 ml of HCl and sterilize.
2. 5.0 mM Tris pH7.5 (1L): 5 ml 1M Tris-HCl pH7.5 (or pH8.0 depending on the subsequent step), add ddH2O to 1L and sterilize.
3. 0.5M EDTA pH8.0 (1L): add 186.1 g Ethylenediaminetetraacetic acid disodium salt dihydrate to 800 ml of ddH20. Since this will solve difficult the use of a magnetic stirrer is advised. Adjust the pH to 8.0 by adding NaOH pellets, as soon as the pH of the solution nears 8.0, the salts will slowly go into solution.
4. TE^−4^ (1L): add 10 ml 1M Tris-HCl pH8.0 to 200 μl EDTA 0.5M pH8.0, to make final concentrations of resp. 10 and 0.1 mM, add ddH2O to 1L and sterilize.
5. Make an 80% ethanol Tris-based buffer by adding 20 ml sterilized 5.0 mM Tris pH 7.5 to 80 ml 100% ethanol. Please note: < 70% buffer will already wash out the bisulfite DNA, resulting in dramatically lower yields.

### Software

Several of the sections in this protocol use R (R Development Core Team, 2011), a free-to-use open-source software for data analysis. Several packages were designed in R with useful tools to evaluate assay designs prior to the measurement on the MassArray system, assess coverage and bisulfite conversion rate and pre-process EpiTYPER® data (RSeqMeth, 2008; MassArray, 2009). One of these, RSeqMeth, requires an older version of R. We added the relevant functions of this package for download on Github, so that they are usable with any R version above 2.13. R scripts can be opened, read and edited using “script editors”: Tinn-R or RStudio™ are both excellent choices since they are free to download and use for non-commercial purposes, have good manuals and a large user base.

1. Install a recent version of R using the CRAN website: http://cran.r-project.org/.
2. Start the latest version of R as administrator (in Microsoft Windows: right click on R in start menu or desktop icon and select “run as administrator”) and run the following lines in R:
  *source (“**http://bioconductor.org/biocLite.R**”)*
  biocLite(“MassArray”)
  install.packages(c(“reshape”,”ggplot2”,”gplots”, “devtools”))
  Select a CRAN repository near to your location, install packages. Of note: R may prompt you to update certain packages. This is often not required for MassArray to work properly, but is advisable to perform.
3. Next, download the files with our custom scripts from Github: https://git.lumc.nl/molepi/EpiTyper.

## Procedures

### Amplicon design

Whether EpiTYPER is suitable for a study depends on the number of regions and samples you wish to measure. One experimental run entails 384 reactions resulting in 126 measurements in triplicate with additional genomic DNA (gDNA) and water control measurements. An EpiTYPER PCR amplicon ranges from 100 to 600 bp, but is typically between 250 and 450 bp. For tips on how to select a region to measure, see Note 1.

After selecting a region of interest, the reference sequence is required as input for assay design software. The sequence of the region of interest can be obtained from various databases. The sequence should be annotated for deviations from the reference sequence (e.g., Single Nucleotide Polymorphisms (SNPs)) that may affect the measurement. If a specific strain of a (model) organism is used, Sanger sequencing of the region of interest can be used to identify deviations from the reference sequence. The steps outlined below are based on the human genome.

1. Browse to the UCSC genome browser http://genome.ucsc.edu/, go to “Genome” and select the most recent human genome build (Kent et al., 2002).
2. Select the region of interest with 300 base pairs upstream and downstream of the target region to allow some flexibility for the assay design software.
3. To control for genetic variation and repeat sequences load the latest common SNP (dbSNP ≥1% of samples, under the “Variation” header) and RepeatMasker tracks (under the “Repeats” header) and set them to “full” view.
4. Go to the “View” tab at the top right of the genome browser and select “DNA”.
5. Press the extended options tab. Highlight the SNPs and repeats. Make sure that each feature is easily and uniquely identifiable.
6. Press “submit” and copy the DNA sequence into a text editor. Go back to the extended options tab and select “reverse complement” and press “submit” again. Copy this sequence into a text editor as well. Having both strands available greatly enhances the design options.
7. Edit your DNA sequence: select your region of interest with [] symbols and place < > around SNPs and repetitive regions. For example:
  AATGGAGTA < **T**>AGAAATAAGG[GCGAGCTACGCGA]TTTGCTCATGG
  The bases between < > will be excluded for primer design. Primer overlap with common SNPs can lead to biased PCR amplifications and artificial bi- and tri-modal DNA methylation distributions. Also, repetitive regions should be excluded in most circumstances. Excluding these regions may increase the reliability of the PCR.
8. Browse to Epidesigner at http://www.epidesigner.com or Methprimer at http://www.urogene.org/cgi-bin/methprimer/methprimer.cgi. Both programs are user-friendly options to design bisulfite primers to amplify a region of interest. An advantage of Epidesigner, is that one can immediately see which CpG sites can be measured with EpiTYPER and design primers for both the forward and reverse strand simultaneously. We here assume that Epidesigner is used. If Methprimer is preferred, copy the amplicon design parameters (annealing temperature etc.) from Epidesigner.
9. Paste the edited sequence in Epidesigner.
10. Remove the check at the “Analyze CpGs in C reaction”. This chemistry will not be used.
11. For “Select strand” choose “both”. This means that primers will be designed for both the forward and reverse strand.
12. Amplicon length may be increased to 600 base pairs.
13. Press “begin”.
14. By hovering with the cursor over the designed amplicons select two amplicons covering the region and/or CpGs of interest for the forward strand (red) and two amplicons for the reverse strand (blue). Please note that spheres in the color red denote CpG dinucleotides that are not covered in the final measurement. Sometimes masses of fragments overlap or are too low or too high to measure on the mass spectrometer. When the software has trouble designing primers please see: Note 2.
15. Export the designs at the bottom of the page as “Order” and as “Detailed”.
16. In the “Order” file rename the primers with suitable and unique identifiers. The small letters in the primer sequence denote tags added for the EpiTYPER biochemistry. They are required for the transcription step, do not remove them.
17. From the “Detailed” file take the genomic sequences for each chosen amplicon and save them in separate tab delimited files using a text editor like notepad. Preferably save the file of each amplicon in a separate directory.
18. Start R (see Software Section of the Materials and Equipment Section).
19. Load in the EpiTYPER R scripts downloaded from Github in R. Run in R:
  source(“directory_of_EpiTYPER.r_supplemental_file/ampliconReport.R”)
20. Perform an *in silico* amplicon prediction. The masses of the fragments generated from the amplified sequence are calculated so that one can assess if enough CpG dinucleotides are covered by different measureable fragments. Run in R:
  ampliconReport()
  If your device has a wider mass range than between 1.5 and 7 kDa you can change the settings to allow a wider mass range:
  *ampliconReport(minMass* = *1500, maxMass* = *7000)*
  Select a tab delimited text file with the genomic sequence of the designed amplicon. The function will write several output files in the directory of this tab delimited file.
  Repeat this this step for each amplicon.
21. In each of the directories of the tab delimited text file with the amplicon sequence, look for the Excel file named after this tab delimited file with the extension: “T Report” and open this file.
22. This file contains the sequences and masses of each so-called “CpG unit”, that is a fragment containing one or multiple CpG sites. The CpG sites are numbered from the 5′ end of the genomic sequence. After each unit there might be a warning message. If the fragment has another (non-) CpG containing fragment within a few Dalton in the mass spectrum a warning is given. These fragments often do not pass the quality control because the closely neighboring fragment causes the variation between triplicate measurements to increase. Furthermore, one may see warnings denoting that there is a “D_” or “OL_” with another CpG unit. This denotes that the masses of either the unmethylated or the methylated CpG unit overlaps with another CpG unit or non-CpG containing fragment. DNA methylation levels from such units cannot be used in downstream analysis.
  Please make sure that enough CpG sites are covered by the amplicon for a productive measurement. If the amplicon does not satisfy your needs design a longer or shorter amplicon or try an amplicon on the other DNA strand for a better result.
23. Some amplicons may also contain a fragment with a TpG and a cytosine. These fragments can be used to estimate the bisulfite conversion for all samples in your study. To automatically check for such fragments start R (see Materials and Equipment) and load the MassArray package in R. Run the following line in R:
  library(MassArray)
24. Open the tab delimited file of the suitable amplicons and copy the sequence. Denote the end and start of your forward and reverse primer with > and < respectively. Run in R:
  *Name_of_amplicon*<*-“paste_the_amplicon_sequence_here”*
  Example:
  Gene1 < - “ATTCCTGGG>ATCGATCGGGAAAATTTCGCGAAAA < GCCCTAATTAA”
25. Then run in R:
  ampliconPrediction(Gene1)
  A figure now appears showing multiple fragmentation profiles of your amplicon. Look at the T(-) fragmentation picture. CpG sites which can be measured are denoted with numbers in blue. Those who cannot be measured are denoted in red. More importantly look for a green fragment. These are fragments that can be used to assess the bisulfite conversion, a key experimental parameter. If your device has a wider mass range than between 1.5 and 7 kDa you can change the settings to allow a wider mass range:
  *ampliconPrediction(Gene1, minMass* = *1500, maxMass* = *7000)*
  Primers designed for bisulfite PCRs have a higher failure rate than normal primers. To save time, one may consider ordering two primer pairs per region of interest. Make sure that at least one region of interest has a fragment suitable to assess the bisulfite conversion efficiency. Use the primer sequences in the “Order” file(s), with the added EpiTYPER specific tags to order your primers. Order HPLC purified PCR primers for optimal results.

### Sample requirements

Pure and high molecular weight gDNA is important for efficient bisulfite conversion and for good EpiTYPER® measurements. The OD260/280 should be between 1.7 and 2.0, lower and higher values may indicate contamination or degradation and the samples may be improved by additional cleaning steps. On a 0.8% agarose gel the gDNA should be high on the gel without a smear and the gDNA should show no additional degradation after a DNAse test (i.e., 16 h incubation at 37 degrees Celsius). The gDNA should also be devoid of RNA by including an RNase step during the DNA extraction. Note that it is important to quantify gDNA using a method that detects double-stranded DNA like Qubit or Picogreen. A spectrophotometric measurement (e.g., using Nanodrop) will generally overestimate the DNA concentration if some degradation is present. For DNA extracted from whole blood with samples this may often be omitted as the resulting gDNA is generally of high quality.

In our hands, extraction of gDNA from other sources than whole blood, especially liver and fat tissue, yielded good results when the gDNA was isolated with the phenol/chloroform method [including a washing step using chloroform:phenol:isoamylalcohol (25:24:1, *v/v*) and a washing step using phenol:isoamylalcohol (24:1, *v/v*)].

### BS-PCR assay validation

To test and optimize the designed bisulfite PCRs (BS-PCR), treat several gDNAs with bisulfite. Also use the same unconverted gDNAs in the PCR tests as negative controls, to exclude amplification of incompletely converted DNA. Perform the PCR optimizations in the same machines and plastics as your final EpiTYPER run.

**Figure 2:**
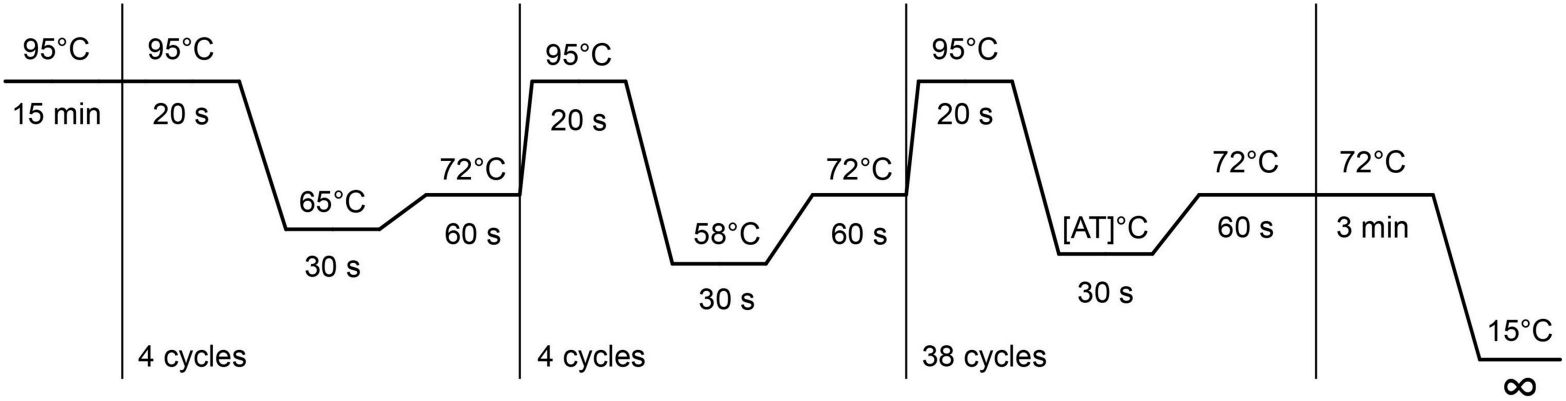
**Step-down bisulfite PCR**.

1. Dilute the primers in a stock solution of 100 pmol/μl and make a working solution at 1 pmol/μl *separately* for the forward and reverse primers. We notice that with time many BS-PCR primer pairs form dimers, resulting in lower PCR efficiency.
2. Create bisulfite treated DNAs by the standard EZ DNA methylation ZymoResearch protocol with a 12 h incubation at 50°C provided with the kit. One can bisulfite treat 10 DNAs per vial of bisulfite. After the protocol measure the concentration of the bisulfite DNA on Nanodrop and dilute to 5 ng/μl. Note that the DNA is now single stranded.
3. Test 6 bisulfite DNAs, 4 genomic DNAs and 2 reactions with water instead of DNA as negative controls with the standard EpiTYPER PCR mix provided in Table 1.
4. Perform the BS-PCR. Incubate the PCR reactions (5 μl) in standard 384-well plates. Carry out the “step-down” PCR thermal cycling protocol (Figure 2) with 15 min at 95°C, followed by 4 cycles: 20 s at 95°C, 30 s at 65°C, and 1 min at 72°C, then followed by 4 cycles: 20 s at 95°C, 30 s at 58°C, and 1 min at 72°C, and subsequently followed by 38 cycles: 20 s at 95°C, 30 s at [AT]°C^*^ and 1 min at 72°C. Carry out the final extension at 72°C for 3 min, and cool down the sample to 15°C. This cycling protocol takes about 2 h. PCR product can be kept at 4°C. Avoid freeze-thaw cycles.
  ^*^The annealing temperature [AT] is amplicon specific. The [AT], optimal annealing temperature is determined by the base pair composition of the primers. Therefore, annealing temperature of the 38-cycle step may vary. Initially start at 53°C for human and 48°C for mouse and rat DNA sources with testing.
5. Visualize 2 μl of PCR product on a 1.5% agarose gel along with an appropriate DNA ladder.
6. A successful PCR amplicon should show a clearly visible band at the correct height without any by-products for all tested bisulfite treated DNAs. Also, there should be no amplification of the gDNA and water controls. Furthermore, check for excessive primer-dimer formation in the water control PCR reactions. If the primer dimer formation is of similar intensity as the BS-PCR product primer dimer formation might become a problem in the final EpiTYPER experiment.
7. For tips on how to optimize your bisulfite PCRs see Note 3.

**Table 1:** **The PCR mix for one PCR reaction**.

	1^*^mix (μl)
MilliQ	0.42
10 × Hotstar Taq PCR buffer	0.50
dNTP mix, 25 mM	0.04
Primermix forward (1 pmol/μl)	1.00
Primermix reverse (1 pmol/μl)	1.00
Hotstar Taq DNA polymerase (5 U/μl)	0.04
Bisulfite-treated DNA (5 ng/ul)/genomic DNA (5 ng/ul) or negative control (MilliQ)	2.00
Total volume	5

### Bisulfite treatment of samples (1 h in the afternoon, overnight, and one morning)

In any quantitative measurement, the occurrence of batch effects is inevitable. Therefore, randomization of samples across bisulfite reactions is recommended. For bisulfite treatment of larger numbers of samples, use of a 96-well based kit will help to reduce batch effects. We recommend the EZ-96 DNA methylation kit by Zymo Research, which entails 12 h incubation at 50°C for the bisulfite reaction. To reduce salts in the final bisulfite treated DNA we made changes to the last wash step and the elution step of this protocol (see below). An alternative protocol with temperature cycles is also provided by Zymo Research, which should result in a higher bisulfite conversion. It is recommended by Illumina for the Illumina 450K DNA methylation arrays, but this protocol will result in additional degradation of the DNA sample, which may result in a lower success rate when measuring longer regions.

For situations with slightly degraded DNA or very little amounts of genomic DNA as starting material one might consider alternatives like the Qiagen Epitect kit. This kit can both handle small amounts of gDNA and also has a protocol for formaldehyde treated samples. We have successfully used this kit to bisulfite treat slightly degraded gDNA.

#### EZ-96 DNA methylation protocol

1. Calculate the amount of gDNA solution which is necessary for 1 μg of gDNA. The maximum volume of gDNA solution that can be used in the bisulfite reaction is 45 μl. Down to 100 ng and up to 2 μg of DNA can be used with this type of column. The manufacturer recommends 500 ng–1.5 μg gDNA for optimal conversion.
2. Follow the EZ-96 DNA methylation protocol, but take extra care of the following points and alterations to the standard protocol.
  - ***Never put*** the bisulfite conversion reaction on the gDNAs ***on ice***.
  - Try to ***minimize the light exposure*** until the completion of the M-Desulphonation step.
  - Preferentially ***prepare the CT conversion reagent directly before*** the intended use. Dissolved CT conversion reagent can only be stored at −20°C for a maximum of a week and only if it is frozen quickly after it is prepared. Be aware that the older the dissolved CT conversion reagent becomes the chance that the bisulfite reaction will be incomplete increases. Keep dissolved CT conversion reagent away from light.
  - ***Replace the second wash step*** after the M-Desulphonation buffer incubation with the Tris-based wash buffer (see Materials and Equipment). This alteration to the protocol is important to minimize the amount of sodium in the converted BS-DNA solution. Sodium will stick to the fragments and shift the mass of your fragments by 22 Dalton, which is very close to the 16 Dalton shift of methylated fragments.
  - ***Replace the last step of the bisulfite protocol***, the EZ-96 DNA methylation Elution step. Incubate the column with 30 μl TE^−4^ for a minimum of 1 min. Centrifuge for 5 min at 2500 × g to elute bisulfite converted DNA. Repeat this step for a 2nd time to obtain 60 μl of eluted BS-DNA. When converting less than 500 ng of gDNA do the first elution with 20 μl and the second with 15 μl.
  - ***Measure the concentration*** of your bisulfite converted DNA (BS-DNA) by UV spectrometry. The 260/280 will be between 1.9 and 2.15 on average. The normal concentration range is: 15–25 ng/μl. Values deviating from these normal ranges are generally caused by low quality gDNA, while much higher yields may be caused by extensive degradation of your sample or ethanol being presents in your BS-DNA. Test samples with deviant values (see Quality control of bisulfite treated samples) or redo the bisulfite conversion. Please note that this concentration is an overestimation of the true concentration because of the degradation during bisulfite treatment.
  - Measure the volume of eluted and concentrated DNA for a subset of samples and calculate the amount of TE^−4^ that is needed to dilute the samples to roughly 5 ng/μl.

Store the BS-DNA at 4°C as repeated freezing and thawing of BS-DNA results in a lower BS-PCR efficiency. Do not store BS-DNA for a prolonged period.

### Quality control of bisulfite treated samples (half a day)

To determine whether the bisulfite treatment was successful and to evaluate how fragmented your bisulfite-treated DNA (BS-DNA) has become, one can perform several test BS-PCR reactions with primers resulting in amplicons of increasing lengths (Table 2). These PCRs are highly specific for BS-DNA and should yield clear, single PCR products on a 1.5% agarose gel (Ehrich et al., 2007). Please be aware that these reactions are for human DNA only. Successful amplification indicates that the BS-DNA is good for subsequent BS-PCR of your amplicons of interest.

1. Choose 3 to 4 quality control BS-PCR primer pairs (Table 2). Choose one primer pair in the lower size ranges (150–250 base pairs) and one primer pair resulting in a PCR amplicon of similar size as your project primer pairs. Select at least one primer pair resulting in an amplicon of a slightly larger size than your project primers.
2. Perform BS-PCR for at least 6 of the samples on a 96-wells bisulfite plate with the test primers using the PCR mix and cycling conditions described above and Table 1. Use an annealing temperature of 53°C for the last step. Include gDNA and water as negative controls.
3. To confirm successful bisulfite treatment and PCR, run 2 μl of the PCR product on a 1.5% agarose gel together with an 100 bp DNA ladder to confirm successful PCR amplification and amplification specificity on the bisulfite treated DNA. No visible PCR product should be visible for the gDNA and water controls.
4. There should be clearly visible BS-PCR products for each size range. This denotes that your BS-DNA is suitable for EpiTYPER measurements.
5. For what to do when (some) samples show no QC BS-PCR products, or only products at the lower size ranges, please refer to Note 4.

**Table 2:** **The primers used for assessing the degradation of your bisulfite treated DNA**.

Assay name^*^	Left primer sequence^**^	Right primer sequence^***^	**Target length**
SQNM_QC_163	TGAGGTGAATTTTAGGGATTGTAGG	ACCTCACAAACTCTCCCAAACC	163
SQNM_QC_176	GTTGAGGGGTAGAGGGAAGTGT	ATCTTCAAACAAAAAAATAACC	176
SQNM_QC_255	TTTTTATTAAAGGTTAAGGTGGTGAT	CAAAACAAAATCCCCACAACC	255
SQNM_QC_328	GGTTTGGGAGAGTTTGTGAGGT	TAACACAAAAAACCCCTTCCTACCA	328
SQNM_QC_362	GTTGAGGGGTAGAGGGAAGTGT	CTCACCAAAAACCAAAATAATAACC	362
SQNM_QC_477	TGTATATGGTTGGGGGTTAGTTG	CCCTCACCAAAAACCAAAATAATAAC	477
SQNM_QC_593	GGTGAATTTTAGGGATTGTAGGGTTTTA	AAACTTCTCCCTCCCAAACCACTAT	593
SQNM_QC_678	AGGGTTTGTTGGGTGATTGGAT	AAACTTCTCCCTCCCAAACCACTAT	678
SQNM_QC_795	GTTTTTTTTAATTGGGGTGGTTT	CTCACCAAAAACCAAAATAATAACC	795
SQNM_QC_960	TTGTTAGATTTTAGATGTTTAAGGTGTTTT	CCTCACCAAAAACCAAAATAATAACC	960

* Primers as originally published by Ehrich et al. (2007).

** Primer 10 bp tag prior to left primer sequence: 5′—AGGAAGAGAG [primer sequence]-3′.

*** Primer 31 bp tag prior to right primer sequence: 5′—CAGTAATACGACTCACTATAGGGAGAAGGCT [primer sequence]—3′.

### Designing your plates (1 h)

Perform all EpiTYPER steps on a bisulfite treated sample in triplicate on a 384 PCR plate. Since measurement are done in triplicate, one can measure up to 126 samples taking into account that some wells are used to add water and unconverted gDNA as PCR controls.

- As mentioned in the section on bisulfite conversion, samples should be randomized. If your project is larger than one 384 plate randomize samples across the 384 plates, preferably with similar proportions of samples from the different bisulfite plates on each 384 plate as to minimize technical batch effects.
- When uploading the sample list to the plate editor software the software will automatically sort the sample list. We advise the following naming convention of your sample list: a number from 001 to 384 denoting the well on the 384 PCR plate, followed by a number unique for each sample (note that the software does not handle text), followed by a number denoting if it is the first, second or third measurement for that sample. These numbers are separated by an underscore. Examples: 001_5186_01; 212_5186_02; 310_5186_03. Well 001–012 should denote the first column on the 384 plate.

Use of this format allows a quick automated creation of the plate in the EpiTYPER software and allows the use of an R script we wrote to automatically perform the data pre-processing (Note 6).

### Performing the EpiTYPER® measurements

The exact EpiTYPER protocol and the required time from start to finish are heavily dependent on the available MassArray setup and may vary. Therefore, this section is restricted to several points and tips that should improve the overall measurement process, independent of the exact set-up. In short, the protocol starts with the bisulfite PCRs on your bisulfite converted samples in triplicate for each PCR amplicon.

- One may consider to first do all steps for one 384 plate, if a large experiment is performed.
- After filling out the gDNA and PCR mix always check for air bubbles. If you find air bubbles gently tap the plate to get rid of them or centrifuge the plate.

After the BS-PCR shrimp alkaline phosphatase is added to nullify the unincorporated DNA nucleotides.

- Directly after this treatment there is opportunity to put 2 μl of roughly 8 samples, the water control and the unconverted gDNA on a 1.5% agarose gel with an appropriate DNA ladder per plate. Place the 384 plate at 4°C temporarily. If one clear PCR product is observed, one can proceed with the rest of the EpiTYPER protocol.

After shrimp alkaline phosphatase treatment the samples are pipetted to a new and clean 384 plate on which the T7 reverse transcription and base specific cleavage is performed.

- Use the remaining BS-PCR product to Sanger sequence a subset of your samples to confirm bisulfite conversion efficiency. For projects with a small number of samples and large number of PCR-reactions it is recommended to Sanger sequence all your samples for at least one amplicon.
- Freeze the left over plate at –20°C for possible additional quality control checks later on.

After T7 reverse transcription and base specific cleavage water will be added. Next, a cleaning resin is added that eliminates salts which may interfere with the mass spectrometer measurement.

- Add sterilized milliQ water to your plate.
- After adding and mixing the resin it is possible to freeze the plate for measurements later on the mass spectrometer. When one wants to (re)measure a frozen plate redo the mixing and spin down of the resin after thawing. But be careful, repeated thaw and freeze cycles will lead to reduced signals.

Now the samples can be transferred to the 384 SpectroCHIP II which is used in the mass spectrometer.

- Test the spot size on a previously used SpectroCHIP II and also assess the spot size on the first SpectroCHIP II used for the new project. Loading too much or too little sample on the SpectroCHIP II will result in noisy measurements and does vary dependent on the ambient temperature and air pressure. The optimum value is dependent on the available set-up and should be provided by the manufacturer's contact person.
- Measure one SpectroCHIP II first and proceed with Data pre-processing and clean up and Note 5, dealing with assessing the quality of the measurement in terms of measurement noise and signal quality, and assess the quality of the measurement before proceeding with the measurement of the entire project.

### Data pre-processing and clean up

After the measurement on the mass spectrometer the data needs to be pre-processed.

1. Open your measurement in the EpiTYPER® Analyser software and select one PCR amplicon.
2. Manually inspect the spectra of multiple samples for irregularities by eye. For details on what to look for see Note 5. Please note that manual inspection of the spectra for measurement noise and peaks not expected based on the amplicon sequence is essential for reliable and high quality EpiTYPER data.
3. Go to “File”, “export grid” and check the boxes at “rotate table data” and “Include comments”. Save the file as “Tab separated values (^*^.txt)” with a unique name and in a unique directory folder. This is the file to perform data pre-processing and quality control on.
4. Exported data for plates with multiple primers will have many NAs for the wells on which another PCR was performed. Furthermore, the triplicate measurements will not be sorted together. An R script is provided to handle the data pre-processing. If you want to use this tool see Note 6. If not, you can do the data cleaning and pre-processing by taking the following steps.
  - Set CpG units of samples with less than two successful measurements for this CpG unit as missing.
  - Set CpG unit measurements with a standard deviation of 0.1 (10% methylation) and larger for a sample as missing.
  - After these two criteria calculate the mean methylation ratio per CpG unit per sample.
  - Remove CpG units with a success rate lower than 80% across samples.
  - Remove CpG units as a whole when the CpG unit has as a warning remark “D” or “OL”. These warnings denote overlaps in mass between other fragments which makes that the DNA methylation signal cannot be assigned uniquely to one CpG unit. Data for such CpG units may be unreliable and is not quantitative.
  - Remove CpG units for which a common SNP is present in the fragment. See the genomic sequence exported from the genome browser (section on Amplicon design).
  - Check the distribution of the DNA methylation data per CpG unit (e.g., by making a histogram). A bi-modal or tri-modal distribution indicates that the measurement of this CpG unit is confounded by a (unknown) SNP in the fragment of the CpG unit. Exclude such a CpG.
5. When performing a project with a limited number of samples Sanger Sequencing of these samples might have indicated that some samples have an incomplete bisulfite conversion, remove such samples. For a larger project Sanger Sequencing of all samples is often not feasible. In such a case perform a qualitative check of the bisulfite conversion of your samples and remove samples for which this qualitative measure indicates an incomplete bisulfite conversion (Note 9).

### Analyzing DNA methylation data

The optimal way to analyze the DNA methylation depends on the project, sample size, and distribution of your DNA methylation data and covariates of interest. Bisulfite conversion plate and also the EpiTYPER experimental plate may be included as covariates in the statistical analysis of larger projects, as they may have an influence on DNA methylation values. For single CpG sites linear regression often suffices. When one wishes to analyze a region as a whole two approaches are often seen in literature. First, the average of the CpG sites can be calculated per sample. This approach causes some information to be lost, but is straightforward in its analysis and interpretation. Linear mixed models (LMMs) may be employed (Loke et al., 2013). These take into account all information on all single CpG units. DNA methylation of adjacent CpG dinucleotides may be correlated and there is also a stronger correlation within a sample. Such correlation structures need to be taken into account to make inferences on statistical significance of regions and LMMs may handle such correlation structures.

## Typical result

Here, we will discuss the design and measurement of an upstream region of the human *Leptin* (*LEP*) promoter. Previously we found that DNA methylation of the *LEP* proximal promoter is associated with various developmental insults (Tobi et al., 2009, 2011; Obermann-Borst et al., 2013). As it is possible that DNA methylation differences effects extend into neighboring regulatory regions (Tobi et al., 2012), it is of interest to explore the larger *LEP* promoter region.

### Design

**Figure 3:**
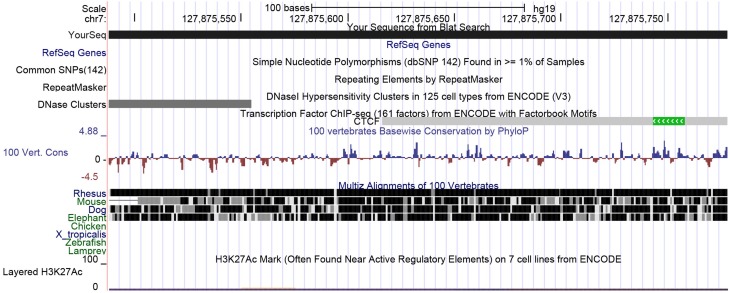
**UCSC view of the *LEP* upstream promoter region spanning 290 bases**. Here a small part of the distal LEP promoter region is shown. The view shows that there are no frequent SNPs nor any repeats in this region. ENCODE data indicates that CTCF may be binding here.

**Figure 4:**
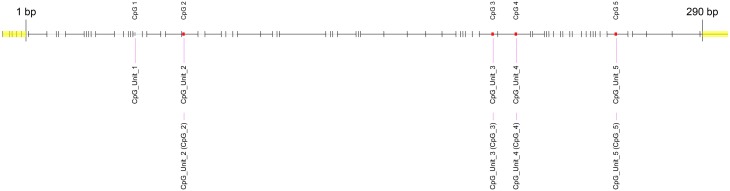
**Fragmentation of *LEP* promoter amplicon**. The fragmentation of the LEP amplicon is given in gray lines. Red dots denote the measureable CpG unit. Clear from the figure is that four out of five CpG unit are measureable and that each CpG dinucleotide is contained in a separate fragment.

First, we used the UCSC genome browser to gain the sequence of the *LEP* promoter, acquiring the first 5 kb upstream of the transcription start site and annotated frequent SNPs and repetitive elements. Using Epidesigner we designed multiple amplicons for the region including one spanning for 290 bases along chr7 and overlapping a *CTCF* binding site according to ENCODE CHIP-seq data (Figure 3). As CTCF binding may be methylation dependent, we took this amplicon further for characterization. This sequence contained no frequent SNPs that could interfere with the reliability of the measurements as shown in Table 3. The amplicon contains 5 CpG sites, of which one cannot be measured because the mass of the CpG unit is too low (Figure 4). The *ampliconPredict* function (RSeqMeth, 2008) indicates that none of the CpG units overlap in mass, have an identical mass, nor are any fragments located within 16 Da of other fragments (Table 4).

**Table 3:** **Upstream sequence of *LEP***.

chr7:127875489-127875778
GAGGTGCTTATGCAAGCAGTCTGGCAAAACACCTCTTTAGGGACAAACGTAGA GCTGGAG
ACTTGGGACGCCTCAGGATGTGTCAGATCACAGCTTTCTGCAGGGCACAAGGC TTCTTGC
TGCTTCTCCAGAACATCTCTCCAAATGCCTGTGCAGCCCTGGGTACCTGGGCA GGGTCCA
GCTCTACAAAGGATCATGGCTCGAGTCCTTCCGCTCCAATGGGAAAGACCAAC CAAGTGA
GAGAAAGAGCATGCCGCCCAGAGGGGGAGCTGGTTGCCATTGTCCTCTTG

**Table 4:** ***ampliconPredict* result for the 290 bp *LEP* amplicon**.

**Name**	**Base mass**	**Desc**	**< 15.9 Da**
CpG_Unit_2	2260.426	CpG_2	NA
CpG_Unit_3	2509.587	CpG_3	NA
CpG_Unit_4	4524.87	CpG_4	NA
CpG_Unit_5	2878.821	CpG_5	NA

This is the prediction given by the EpiTYPER.r script taken from the RSeqMeth package. Four out of 5 CpGs are measured in separate fragments. The mass of the fragments range from 2260 Da till 4525 Da and no other fragments are located with 15.9 Da, indicating that the measurement will not be influenced by the signal for another fragment. Also no warnings are given of possible overlaps between CpG units. Also no CpG unit has the same mass as another.

### BS-PCR validation of *LEP*

Next, we tested the PCR of this amplicon using 8 test gDNA samples that were extracted from whole blood and bisulfite converted as described earlier. We included additional controls, namely non-converted genomic DNA and several PCR reactions with water added instead of gDNA. One clear band was observed on a 1.5% agarose gel (*data not shown*), but the intensity of the band was not that strong. Therefore, the PCR was redone with a 3°C lower annealing temperature (50°C) in the last step of the step down protocol PCR protocol. This yielded a strong and still unique PCR product on gel (*data not shown*) and we chose to measure this amplicon on a small number of samples using EpiTYPER.

### Measurement of *LEP* with EpiTYPER

Ten samples, one non-bisulfite converted gDNA and one PCR with water added instead of gDNA were measured in triplicate on a 384 plate.

**Figure 5:**
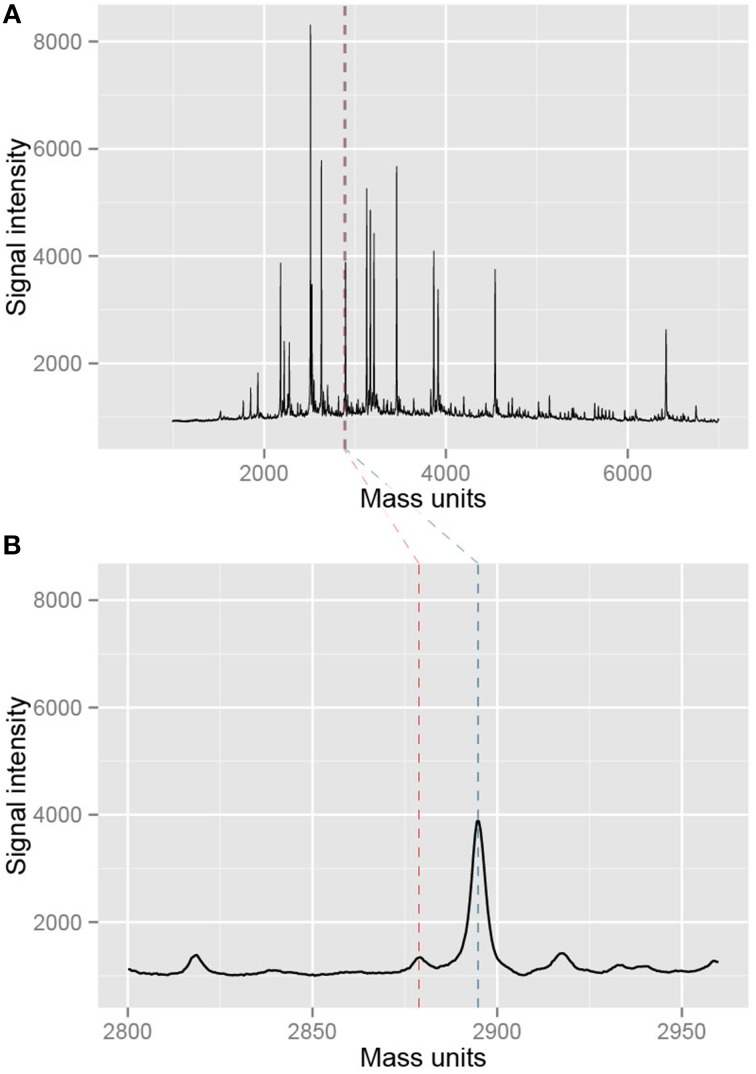
**Example of mass spectra resulting from the measurement with EpiTYPER**. **(A)** Shows an entire mass spectrum of a single measurement of a sample. Black peaks are measured fragments while the blue and red dotted lines denote a selected CpG fragment in the EpiTYPER software. This particular part of the mass spectrum is shown in **(B)**. The spectrum is nice and smooth, indicating a good quality of the measurement.

First, we inspected at the entire mass spectrum of several of these 12 triplicate measurements. The measurements all showed a nice baseline following the x-axis (Figure 5A), indicative of little measurement noise, and the signals of the predicted peaks are high. Moreover, additional peaks were distributed randomly across the measurements and lower in intensity than the predicted peaks. Together, this is indicative for optimal spotting and laser intensity settings, little unspecific cleavage and no non-specific PCR product formation (see Note 5). When looking at some CpG units in more detail the spectra were smooth (Figure 5B), again indicative of a good quality measurement.

Next, we exported the raw data from the EpiTYPER software and found that the EpiTYPER software has given a warning for the CpG site that cannot be measured (Table 5); its mass is too low to be measured. For CpG unit 3 an additional remark is given, namely “SN3”, indicating that a non-CpG fragment overlaps the unmethylated CpG unit 3 fragment. The EpiTYPER software is often successful in subtracting the signal of such fragments from the methylation measurement. Nonetheless, such units often have a higher measurement error. Also in this case the standard deviation of the triplicate measurement is high and only 3 out of 10 samples have an observation that passes the QC filtering steps (Table 5). All other 3 CpG units however, have a high quality measurement and can be used for analysis purposes.

**Table 5:** **Results for the LEP amplicon measurement**.

**Standard deviation**	**CpG_1**	**CpG_2**	**CpG_3**	**CpG_4**
**Warning**	**L_mass**	**NA**	**SN3**	**NA**
**Sample**				
1	NA	0.025	0.103	0.010
2	NA	0.012	0.076	0.000
3	NA	0.012	0.119	0.010
4	NA	0.015	0.248	0.006
5	NA	0.010	0.025	0.006
6	NA	0.067	0.319	0.055
7	NA	0.021	0.154	0.006
8	NA	0.035	0.112	0.006
9	NA	0.015	0.020	0.006
10	NA	0.026	0.140	0.012
gDNA	NA	NA	NA	NA
water	NA	NA	NA	NA
**Filtered Methylation values**	**CpG_1**	**CpG_2**	**CpG_3**	**CpG_4**
**Sample**				
1	NA	0.713	NA	0.930
2	NA	0.813	0.487	0.930
3	NA	0.783	NA	0.930
4	NA	0.817	NA	0.937
5	NA	0.790	0.897	0.923
6	NA	0.757	NA	0.903
7	NA	0.797	NA	0.927
8	NA	0.807	NA	0.937
9	NA	0.787	0.880	0.933
10	NA	0.830	NA	0.937
gDNA	NA	NA	NA	NA

The standard deviation and filtered final methylation values (ratio) is given. CpG unit 1 has a low mass and cannot be measured (data is all missing: NA) while CpG unit 3 has a warning message (“SN3”) denoting a non-CpG containing fragment overlapping the unit. This results in an overall higher technical variability between the triplicates, resulting that only 3 out of 10 samples have a measurement passing the QC steps.

## Notes

### Note 1. advice on where to measure

In many instances one starts with a gene or set of genes. The genomic regions in such instances are too large to assess in their totality and it is currently largely unknown which CpG sites have a regulatory potential. For some genes a thorough literature search may yield some insights. However, for most genes little is known.

ENCODE and Roadmap Epigenetics data can be used to select regions of interest. The popular UCSC and ENSEMBL genome browser are not always up to date with the latest ENCODE and Roadmap data. ENCODE has an excellent UCSC portal by which many epigenetic datasets can be visualized. The Roadmap project also has a dedicated browser. All links can be found at: http://www.roadmapepigenomics.org/. ENCODE has an “experiment matrix”, while the roadmap has a similar “epigenome atlas” containing overviews of the available data types and datasets. The data is continually evolving as both projects continue. Therefore, we limit ourselves to some general tips on which type of data might be a good starting point in your effort to prioritize regions for EpiTYPER measurements.

- One may start by visualizing tracks containing DNA methylation data in tissues and cell lines relevant for the planned study. Regions exhibiting variation between tissues and cell types may be prioritized.
- Both projects provide so-called chromatin states for multiple cell lines and tissues, which can be used to find active (alternative) promoters and enhancers in cell types of interest. In the regular UCSC genome browser the chromatin states for the 8 main ENCODE cell lines can be found under “Regulation” and the “ENCODE Histone Modification Tracks” tab. Chromatin states for more and more cell types are becoming available.
- Transcription factor CHIP-seq data is also a good start. Are there any transcription factors that are DNA methylation sensitive and overlapping with interesting HMM chromatin promoter and enhancer states in and around your gene of interest? Examples of DNA methylation sensitive transcription factors include NFkβ, CTCF, MBD2, and NGF1-A. Predicted binding sites might likewise be useful to prioritize regions to measure.
- Another indication of functionality can be inferred by extracting DNA methylation levels for multiple cell types in certain regions and correlate the DNA methylation levels with normalized RNA seq data of neighboring genes in the same cell types. Note that data may not be generated using the same cell culture experiment or donor.

### Note 2. problems with designing a bisulfite PCR amplicon

Sometimes it is difficult to design primers for some regions. The following changes from the standard protocol may be useful.

- Set the minimum primer size to 16 basepairs and try to design primers.
- Secondly set the maximum amplicon size to 650 basepairs. Several groups have worked with amplicons larger than 500 base pairs. However, good quality input gDNA is required.
- Remove the check at “maximize coverage” and lower the “Product CPGs” option.
- As a last resort, set the number of primer “Non-CpG ‘C’s” to 3. However, test the specificity for bisulfite converted DNA more rigorously for these amplicons, by testing more genomic DNAs (see BS-PCR assay validation).

### Note 3. optimizing your bisulfite PCRs

The bisulfite PCR is an important parameter in the EpiTYPER® protocol. A low amount of product will result in an unfavorable signal-to-noise ratio.

- If the BS-PCR gives a weak product and/or some weak by-products, try a higher temperature in the first two steps, followed by a lowered annealing temperature in the final cycling step. Start by increasing your first two annealing temperatures by 2 degrees and decreasing your final annealing temperature by 3 degrees.
- The amount of a weak unique product can be increased by using more primer and more taq polymerase (up to 50%). Adding more BS- DNA only helps when the BS-DNA was rather degraded. Extra PCR cycles will not be very effective in increase the amount of product, since the number of cycles is already high.
- Multiple strong PCR products, re-design primers.
- No product at all, try to use much lower annealing temperatures or re-design primers.

### Note 4. negative outcomes of bisulfite quality control

If no or little product is found in the test BS-PCR reactions, it is likely that the BS-DNA is not suitable. The most likely cause is that your input-DNA was already slightly degraded or dirty. Another common cause is that an error was made during the bisulfite protocol. Some additional considerations are now necessary.

- When all size ranges failed, re-assess the sample purity of the samples for which these test BS-PCRs failed. We found that it may help to perform another protein K treatment and traditional chloroform DNA clean-up before re-doing the bisulfite treatment.
- Most often only test BS-PCR reaction of the larger size ranges fail. This often indicates extended degradation of your samples during bisulfite treatment. If the amplicons in your project are smaller than the failed QC BS-PCR, your measurements may still be successful. However, since failed QC BS-PCRs may also point to a failed bisulfite conversion it is advisable to proceed with caution and first perform Sanger sequencing of the bisulfite converted DNA to confirm complete conversion for a subset of the samples (on the 96-wells plate) before proceeding with the EpiTYPER measurements. If your amplicons of interest are of a similar size range as the failed test BS-PCRs it will be necessary to re-do the bisulfite treatment. A bisulfite kit able to handle slightly degraded DNA, like the Qiagen Epitect kit with FFPE protocol might be considered as an alternative in such cases.

### Note 5. inspecting EpiTYPER® mass spectra

It is important to manually inspect a proportion of the generated EpiTYPER spectra in the Analysis software to identify possible issues with your biochemistry, mass spectrometer, or automated liquid handlers.

- Open the EpiTYPER® software and check that the “T reverse” tab is activated.
- Set the “Spectrum View” tab on “recalibrated.”
- Look at multiple samples. Does the baseline of the spectrum run parallel with the x-axis? The vast majority of samples should have a low baseline. If lots of samples have arched shaped baselines there might be something wrong with the laser intensity or the spotter. Arched shaped spectra will negatively influence the quality of the measurement by inflating the background signal, especially in the lower mass ranges.
- In the “View” panel go to “Enable/Disable Pane”, check “additional Peaks”. In the panel below an additional tab now appears. Look at multiple samples. Go through the additional peaks and confirm that they are mostly not larger than the background signal. Normally roughly 10–30 additional peaks are called by the software. If high signal intensity additional peaks are found in many samples there might be an unspecific BS-PCR product in your reaction. Double check the PCR amplicon sequence file to make sure the correct sequence was used. Also, place some BS-PCR reactions on gel to validate to no non-specific PCR products are confounding the measurements.
- Go to a sample and zoom in to a level that roughly 4–8 individual peaks can be discerned. Are the peaks and background baseline nice and smooth? If a significant proportion of your samples show a very jagged baseline and peaks that do not have a smooth curve there might be something suboptimal in the mass spectrometer settings or the SpectroCHIP II. Consult with the manufacturer to verify the mass spectrometer settings are still optimal.
- Zoom in to the lower mass ranges (1000–2000 Da) and higher mass ranges >7000 Da. Normally some variation is seen in outer mass ranges, indicating a slightly less optimal signal-to-noise ratio. Especially in the higher mass ranges the optimal detection cut-off may vary depending on the conditions during the spotting of the sample on the mass spectrometry plates. When the baseline becomes jagged and many additional peaks are called by the EpiTYPER software changes in the spotting conditions and/or mass spectrometer are needed.
- Look at samples with some missing values. Zoom in to the missing CpG units in the spectrum. Are they indeed missing peaks, or are the peaks low compared to the background? Lots of missing peaks might indicate that not enough BS-PCR product was formed or that the transcription step went suboptimal. It is good to try to discern if there is a pattern to such samples: are the samples located next to each other on a 384 plate? In such case there might be a bad pipetting channel in one of your automated liquid handlers. If not, one might need to place some BS-PCRs on agarose gel and assess if it might be prudent to re-do the measurement.

### Note 6. running the R data cleaning and pre-processing script.

To aid data pre-processing, we provide an R script that handles the data pre-processing and data cleaning.

- Scripts are available in GitHub using the following link: https://git.lumc.nl/molepi/QC_Epityper.
- Set your R working directory to the location of your exported DNA methylation data, have one unique directory with all DNA methylation data from all plates belonging to a particular project. Run the following line in R:
  setwd(“C:/directory_with_DNA_methylation_data”)
- Load the scripts downloaded from Github to R by referring to the file as follows, run in R:
  source(“C:/location_of_Supplemental_file/EpiTYPER.r”)
- Define samples you wish to exclude from analysis. For instance, the genomic DNA and negative controls. Run the following line in R, denoting any samples you wish to exclude with brackets:
  *Donots*<*- c(“negative_control”, “gDNA1”, “gDNA2”)*
- Define the directory where the script can find all relevant EpiTYPER data for one project. Run the following line:
  *directory*<*- getwd()*
- Do the pre-processing and QC. If you want to remove CpG units with masses overlapping with other fragments set “remove = T”, otherwise: “remove=F” If you want to set another cut-off for the CpG units please change “succesrate = 0.8” (successrate > 80%) to the required threshold. Remove samples by adding the variable with the samples to exclude to the “sampRemove” argument, if no samples need to be removed set “sampRemove=NULL”. Run in R, with the possible desired edits:
  QC.Epityper(directory, succesrate=0.8, remove=TRUE, sampRemove=donots)
- An “output” directory is automatically made in the directory of your DNA methylation data. A tab delimited text file is created for further analyses for each amplicon. The script will also have created PDF files with success rates and other metrics per PCR amplicon in the same directory.

### Note 7. qualitative assessment of bisulfite conversion

For a large project it is not feasible to do an assessment of the bisulfite conversion rate for all samples with Sanger Sequencing. After having confirmed the bisulfite conversion for a subset of your samples by Sanger Sequencing it is possible to get a qualitative assessment for each sample if you have a PCR amplicon with a so-called conversion control fragment. An additional data export from the EpiTYPER software is required to perform bisulfite conversion assessment using the MassArray package. For additional info and more details please refer to the Bioconductor MassArray page (on http://www.bioconductor.org/ search for “MassArray”).

- Make sure to have the genomic sequence of your amplicon with conversion control available. Verify that no common SNP is present in this conversion fragment. Use the latest common, ≥1% of samples, dbSNP version to this end.
- Open the EpiTYPER® Analysis software.
- Load one amplicon of your plate of interest. This script can only be performed per amplicon and per individual 384 plate.
- Once you have opened an individual amplicon in the EpiTYPER® application, enable display of both the “T reverse” and “C reverse” tabs.
- Then go to “View”, “Enable/Disable Pane”, make sure that both the “Show All Matched Peaks” and “Show All Missing Peaks” options are checked.
- Go to “File”, “Export Grid” and save the tab delimited file in a unique directory with a unique name.
- Open R and run the script:
  library(MassArray)
- Annotate your amplicon sequence with the start and end of your forward and reverse primers using > and < respectively.
  Example:
  Gene1 < - “ATTCCCAAAT>AATTTACGCGAATGGGGCCGCG < TAACCAACCTTTGG”
  Enter your amplicon in R and run the script:
  *amplicon*<*- “paste_sequence_of_your_amplicon_here”*
- Set the work directory of R to the work directory of your exported tab delimited file by running the following lines in R:
  directory_name=“C:/Directory_name_of_exported_file”
  setwd(directory_name)
- Then processes your data, and run:
  *data*<*- new(“MassArrayData”, amplicon, “Name_of_Exported_file.txt”)*
  Depending on your computer this may take a considerable amount of time.
- Now determine your bisulfite conversion. Run:
  *bisulfite* <*- lapply(data, slot, “quality.conversion”)*
  *conversion* <- 100^*^as.numeric(lapply(bisulfite, mean, na.rm=TRUE))
- The resulting vector of numbers is the average bisulfite conversion per well in the 384 plate. The value denoted should be < 2 per measurement. Higher values may denote a sample with an incomplete bisulfite conversion or some amplification of unconverted DNA. For some samples no assessment (NA) can be made. In most cases a high background signal around the conversion control fragment is the cause. The order of samples is the same as the order in the EpiTYPER® Analysis software. Please note that this assessment of bisulfite conversion is a qualitative measure, not an exact measure, of the bisulfite conversion. The software is looking for a fragment that is inherently low in signal intensity. The signal-to-noise ratio is therefore always very unfavorable.
- The vector can be saved as tab delimited text file by running:
  write.table(conversion, file=“desired_name_of_file.txt”, col.names=F, row.names=F, sep=“\t,” quote=F)

### Conflict of interest statement

The authors declare that the research was conducted in the absence of any commercial or financial relationships that could be construed as a potential conflict of interest.
